# The relationship between moral sensitivity and prosocial behavior in college students: the mediating roles of moral disengagement and reciprocity norms

**DOI:** 10.3389/fpsyg.2024.1508962

**Published:** 2025-01-15

**Authors:** Zhiyong Li, Danqi Wang, Junhe Liao, Zhibin Jin

**Affiliations:** ^1^School of Education, Huainan Normal University, Huainan, China; ^2^School of Education, Huaibei Normal University, Huaibei, China

**Keywords:** moral sensitivity, prosocial behavior, moral disengagement, positive reciprocity, negative reciprocity

## Abstract

**Introduction:**

This study examined the relationship between moral sensitivity and prosocial behavior, as well as the mediating role of moral disengagement and reciprocity norms.

**Methods:**

Totally 567 college students completed Dispositional Moral Sensitivity Questionnaire, Moral Disengagement Scale for Chinese Students, Reciprocity Norm Scale, Prosocial Tendencies Measure for Adolescent.

**Results:**

The results showed that, moral disengagement and reciprocity norms played multiple mediating roles between moral sensitivity and prosocial behavior. Specifically, moral sensitivity affected prosocial behavior through the independent mediating effect of moral disengagement, reciprocity norms, as well as the chain mediating effect of moral disengagement and reciprocity norms.

**Discussion:**

In summary, this study reveals the mechanism underlying the relationship between moral sensitivity and prosocial behavior, which has certain theoretical and practical significance for promoting college students’ prosocial behavior from the perspective of moral sensitivity.

## Introduction

Throughout the history of psychology, researchers have always paid close attention to prosocial behavior in their exploration journey, as it highlights the brilliance of human nature when dealing with core psychological issues such as the relationship between oneself and others, oneself and groups, oneself and society in social life, as well as the interests of present and future, material and spiritual, individual and collective (Zhang and Kou 2019). It is crucial not only for maintaining positive interpersonal relationships but also for fostering a benevolent social mindset (Liu et al., 2022). Prosocial behavior encompasses actions by individuals in social interactions that adhere to social expectations and benefit others, groups, and society (Piff and Robinson, 2017). As the foundational element of moral behavior, it significantly impacts the occurrence of prosocial actions (Miles and Upenieks, 2021).

Moral sensitivity is the ability to recognize and interpret moral issues based on one’s experiences and understanding, specifically identifying when a situation presents a moral dilemma. It is a key component of moral cognition (Gao and Mao, 2024). This sensitivity involves perceiving the moral significance of various aspects of a situation, including interpreting others’ reactions and feelings, understanding causal chains and their impacts, and employing empathy and role-taking skills (Reynolds and Miller, 2015). Research shows that individuals with high moral sensitivity are more likely to engage in prosocial behaviors (French et al., 2018). Thus, moral sensitivity is crucial for the development of prosocial behavior, although the specific mechanisms through which it influences prosocial actions remain unclear. According to the four-component model of moral psychology, as individuals’ moral judgment, identity, and motivation evolve, the activation of moral sensitivity becomes increasingly indirect (Thoma and Bebeau, 2013). Moral disengagement and reciprocity norms are potential mediators in the relationship between moral sensitivity and moral behavior.

Moral disengagement refers to a cognitive mechanism that allows individuals to avoid self-sanction by disconnecting their actions from their internal moral standards (Schaefer and Bouwmeester, 2020). This process is crucial in both moral and immoral behavior (Jean et al., 2012). According to moral disengagement theory, while experiential self-regulation can typically suppress undesirable behavior, moral disengagement selectively renders self-regulation mechanisms ineffective, leading to a lack of guilt following moral transgressions (Gutzwiller-Helfenfinge, 2015). Empirical studies have shown that moral disengagement negatively predicts prosocial behavior; individuals with higher tendencies toward moral disengagement tend to exhibit lower prosocial tendencies (Killer et al., 2019). When individuals perceive that moral behavior involves personal costs or sacrifices, they may employ defensive strategies such as moral disengagement or reinterpretation of moral situations to justify alternative actions (Zhao and Kushnir, 2023). Thus, moral disengagement may mediate the effect of moral sensitivity on prosocial behavior. Reciprocity norms are prevalent in human societies and manifest across various social relationships, setting moral standards for giving and receiving help and forming fundamental principles in social exchanges (Guala, 2012).

Conversely, negative reciprocators are attuned to negative behaviors, remembering harm and retaliating with negative actions—returning resentment for resentment (Schug et al., 2016). Social exchange theory suggests that individuals internalize these norms to maintain balance in interpersonal relationships and psychological states, influencing whether they exhibit moral or immoral behavior (Jeung et al., 2017). Research shows that positive reciprocators are more likely to experience positive emotions like gratitude after receiving help, which fosters prosocial behavior (Leimgruber et al., 2014). In contrast, negative reciprocity is linked to negative emotions such as anger, which can reduce prosocial behavior (Wu et al., 2014). Given that moral sensitivity involves complex interactions between moral cognition and emotions (Zhou et al., 2024), individuals with high moral sensitivity are more likely to follow reciprocity norms, displaying corresponding positive or negative reciprocity behaviors (Van Baar et al., 2019). Thus, reciprocity norms may mediate the effect of moral sensitivity on prosocial behavior. Furthermore, moral disengagement may influence these norms. As a self-regulation process, individuals with high moral disengagement might adopt reciprocity norms such as “returning good for good and resentment for resentment” to alleviate the tension from not engaging in moral behavior, thus avoiding self-sanction (Jean et al., 2012). Consequently, moral disengagement might reduce positive reciprocity while increasing negative reciprocity. Therefore, it can be hypothesized that moral disengagement and reciprocity norms may serve as chain-mediators in the relationship between moral sensitivity and prosocial behavior.

In summary, this study aims to explore the impact of moral sensitivity on prosocial behavior and the multiple mediating effects of moral disengagement and reciprocity norms based on the four-component model theory of moral psychology. The following hypotheses are proposed: H1: Moral sensitivity is significantly positively correlated with prosocial behavior; H2: Moral disengagement mediates the relationship between moral sensitivity and prosocial behavior; H3: Reciprocity norms mediate the relationship between moral sensitivity and prosocial behavior; H4: Moral disengagement and reciprocity norms play a chain-mediating role in the relationship between moral sensitivity and prosocial behavior.

## Methods

### Participants

A convenience sampling method was adopted to recruit college students in Anhui Province, China. The participants completed a survey anonymously in a classroom. Instructions clearly indicated that the survey included a lie detection scale to ensure that the participants responded carefully. 630 students from two colleges participated in the survey, with 567 valid responses collected, yielding a response rate of 90.00%. The sample comprised 102 males and 465 females, with 200 participants from urban areas, and 367 from rural areas. Participants’ ages ranged from 17 to 23 years (M = 18.94, SD = 0.90).

This study was approved by the Academic Committee of the School of Education of Huainan Normal University, Anhui, PR China. All participating Chinese college students signed an informed consent form. The informed consent form includes information on the purpose and procedures of the study, confidentiality agreement, voluntary participation principle, as well as control for variables such as gender and grade of the subjects.

### Measures

#### Moral sensitivity

The Dispositional Moral Sensitivity Questionnaire (DMSQ) by Zheng and Cen (2008) was used. It includes 28 items divided into five factors: empathic guilt, punishment tendency, empathic worry, awareness frequency, and empathy phenomenon. Responses are measured on a 5-point Likert scale (1 = Strongly Disagree to 5 = Strongly Agree), with higher scores indicating greater moral sensitivity. In this study, the Cronbach’s alpha coefficient of the scale was 0.84, and the validated factor analysis fit index was good, indicating that the questionnaire had good reliability and validity in this study.

#### Moral disengagement

Chinese version of Moral Disengagement Scale by Wang and Yang (2010) was utilized. This 26-item questionnaire is divided into eight factors: moral justification, euphemistic labeling, advantageous comparison, diffusion of responsibility, displacement of responsibility, distortion of consequences, dehumanization, and attribution of blame. Responses are measured on a 5-point Likert scale (1 = Strongly Disagree to 5 = Strongly Agree), with higher scores indicating higher levels of moral disengagement. In this study, the Cronbach’s alpha coefficient of the scale was 0.80, and the validated factor analysis fit index was good, indicating that the questionnaire had good reliability and validity in this study.

### Reciprocity norm

The Reciprocity Norm Questionnaire developed by Eisenberger et al. (2004) was employed. It includes 24 items, categorized into two factors: positive and negative reciprocity norms. Responses are measured on a 7-point Likert scale (1 = Strongly Disagree to 7 = Strongly Agree), with higher scores reflecting stronger tendencies towards positive or negative reciprocity. In this study, the Cronbach’s alpha coefficient of positive reciprocity, negative reciprocity, and reciprocity norm were 0.84, 0.87, and 0.85, respectively. The validated factor analysis fit index was good. This indicated that the questionnaire had good reliability and validity in this study.

### Adolescent prosocial tendencies

The Adolescent Prosocial Tendencies Scale was used by Kou et al. (2007). It includes 26 items across six factors: public, anonymous, altruistic, compliant, emotional, and emergency prosocial tendencies. Responses are rated on a 5-point Likert scale (1 = Not Very Not Like Me to 5 = Very Like Me), with higher scores indicating higher prosocial tendencies. In this study, the Cronbach’s alpha coefficient of the scale was 0.90, and the validated factor analysis fit index was good, indicating that the questionnaire had good reliability and validity in this study.

### Statistical methods

Descriptive statistics, correlation analysis, and multiple mediation effect analyses were conducted using SPSS 25.0. Structural equation modeling and further examination of multiple mediation effects were performed using Amos 25.0.

### Check for common method bias

This study adopts Harman’s one-factor test (Zhou and Long, 2004) to examine common method biases. Unrotated factor analysis showed that 33 factors were generated, and the first principal factor explained 11.14% of the variance, which is less than 40%, indicating that there was no serious common method bias in this study.

## Results

### Descriptive statistics and correlation analysis

Descriptive statistics and Pearson correlation analysis were conducted for moral sensitivity, moral disengagement, reciprocity norms, and prosocial behavior (see Table 1). The results revealed that moral sensitivity was significantly positively correlated with moral disengagement, positive reciprocity, negative reciprocity, and prosocial behavior. Moral disengagement was significantly negatively correlated with positive reciprocity and prosocial behavior, and significantly positively correlated with negative reciprocity. Prosocial behavior was significantly positively correlated with positive reciprocity and negatively correlated with negative reciprocity. The relationship between the variables supports the subsequent hypothesis test, which provides a better foundation for the mediation effect test in this study.

**Table 1:** Correlation analysis (*n* = 567).

	Variable	*M ± SD*	1	2	3	4	5
1	Moral sensitivity	87.93 ± 12.09	1				
2	Moral disengagement	52.04 ± 9.75	0.12^**^	1			
3	Positive reciprocity	63.16 ± 5.48	0.32^**^	−0.15^**^	1		
4	Negative reciprocity	53.97 ± 13.09	0.25^**^	0.34^**^	0.10^*^	1	
5	Prosocial behavior	95.28 ± 11.59	0.29^**^	−0.22^**^	0.36^**^	−0.21^**^	1

^**^p < 0.01; ^*^p < 0.05.

### Multiple mediation analysis

Multiple mediation effects were examined using the PROCESS macro for SPSS developed by Hayes (2012), with 5,000 bootstrap samples and 95% confidence intervals. Based on the correlation analysis, two mediation models, M1 and M2, were constructed. Model M1 includes the following pathways: moral sensitivity → prosocial behavior; moral sensitivity → moral disengagement → prosocial behavior; moral sensitivity → positive reciprocity → prosocial behavior; and moral sensitivity → moral disengagement → positive reciprocity → prosocial behavior. Model M2 mirrors Model M1, except that positive reciprocity is replaced by negative reciprocity.

### Examination and analysis of model M1

As shown in Table 2, moral sensitivity significantly and positively predicted both moral disengagement (*β* = 0.12, *p* < 0.001) and prosocial behavior (*β* = 0.29, *p* < 0.001). When moral sensitivity and moral disengagement were used to predict positive reciprocity, moral sensitivity was a significant positive predictor (β = 0.34, *p* < 0.001), whereas moral disengagement was a significant negative predictor (β = −0.19, *p* < 0.001). In the model where moral sensitivity, moral disengagement, and positive reciprocity were used to predict prosocial behavior, both moral sensitivity (β = 0.23, *p* < 0.001) and positive reciprocity (β = 0.26, *p* < 0.001) were significant positive predictors of prosocial behavior. Conversely, moral disengagement significantly negatively predicted prosocial behavior (β = −0.21, *p* < 0.001). These results suggest that moral disengagement and positive reciprocity partially mediate the relationship between moral sensitivity and prosocial behavior.

**Table 2:** Regression analysis of variable relationships in model M1.

Dependent variable	Independent variable	*R*	*R^2^*	*F*	*β*	*t*	*Boot LLCI*	*Boot ULCI*
Prosocial behavior	Moral sensitivity	0.29	0.08	51.12^***^	0.29	7.15^***^	0.21	0.37
Moral disengagement	Prosocial behavior	0.12	0.01	7.57^**^	0.12	2.75^**^	0.03	0.20
	Moral disengagement	0.34	0.13	43.75^***^	−0.19	−4.76^***^	−0.26	−0.11
Positive reciprocity	Prosocial behavior				0.34	2.55^***^	0.26	0.41
	Moral disengagement	0.45	0.21	48.68^***^	−0.21	−5.41^***^	−0.28	−0.13
Prosocial behavior	Positive reciprocity				0.26	6.40^***^	0.18	0.34
	Prosocial behavior				0.23	5.74^***^	0.15	0.31

^***^p < 0.001, ^**^p < 0.05; ^*^p < 0.01.

### Mediation effects analysis

Table 3 shows that the total mediation effect of moral sensitivity on prosocial behavior is 0.06, with a Bootstrap 95% confidence interval that does not include zero, indicating significant mediation by moral disengagement and positive reciprocity. This total mediation effect comprises three indirect pathways: Moral Sensitivity → Moral Disengagement → Prosocial Behavior (effect size: −0.02; 95% CI: [−0.05, −0.006]); Moral Sensitivity → Moral Disengagement → Positive Reciprocity → Prosocial Behavior (effect size: −0.01; 95% CI: [−0.01, −0.001]); Moral Sensitivity → Positive Reciprocity → Prosocial Behavior (effect size: 0.09; 95% CI: [0.06, 0.12]). The confidence intervals of the tree pathways does not include zero, indicating a significant effect.

**Table 3:** Mediating effects analysis of moral disengagement and positive reciprocity.

Pathway	Effect size	SE	95% CI
Total mediating effect	0.06	0.02	[0.02, 0.10]
Moral sensitivity → moral disengagement → prosocial behavior	−0.02	0.01	[−0.05, −0.006]
Moral sensitivity → moral disengagement → positive reciprocity → prosocial behavior	−0.01	0.003	[−0.01, −0.001]
Moral sensitivity → positive reciprocity → prosocial behavior	0.09	0.02	[0.06, 0.12]

### Examination and analysis of model M2

As shown in Table 4, both moral sensitivity and moral disengagement significantly and positively predicted negative reciprocity (*β* = 0.22, *p* < 0.001; β = 0.31, *p* < 0.001). When moral sensitivity, moral disengagement, and negative reciprocity were used to predict prosocial behavior, moral disengagement and negative reciprocity significantly and negatively predicted prosocial behavior (β = −0.19, *p* < 0.001; β = −0.25, *p* < 0.001). In contrast, moral sensitivity significantly and positively predicted prosocial behavior (β = 0.37, *p* < 0.001). These results indicate that moral disengagement and negative reciprocity partially mediate the relationship between moral sensitivity and prosocial behavior.

**Table 4:** Regression analysis of variable relationships in model M2.

Dependent variable	Independent variable	*R*	*R^2^*	*F*	*β*	*t*	95%CI
Prosocial behavior	Moral sensitivity	0.29	0.08	51.12^***^	0.29	7.15^***^	[0.21,0.37]
Moral disengagement	Moral sensitivity	0.12	0.01	7.57^**^	0.12	2.75^**^	[0.03, 0.20]
	Moral disengagement	0.40	0.16	53.44^***^	0.31	7.98^***^	[0.23, 0.39]
Negative reciprocity	Moral sensitivity				0.22	5.62^***^	[0.14, 0.30]
	Moral disengagement	0.45	0.20	46.53^***^	−0.19	−4.52^***^	[−0.26, −0.10]
Prosocial behavior	Negative reciprocity				−0.25	−5.96^***^	[−0.33, −0.17]
	Moral sensitivity				0.37	9.51^***^	[0.30, 0.45]

^***^p < 0.001, ^**^p < 0.05; ^*^p < 0.01.

### Mediating effect analysis for model M2

Table 5 reveals that the total mediation effect of moral sensitivity on prosocial behavior is −0.08, with a Bootstrap 95% confidence interval that does not include zero, indicating significant mediation by moral disengagement and negative reciprocity. This total mediation effect includes three indirect pathways: (effect size: −0.02; 95% CI: [−0.05, −0.01]); (effect size: −0.01); moral sensitivity → moral disengagement → negative reciprocity → prosocial behavior (effect size: −0.01; 95% CI: [−0.09, −0.003]); Moral Sensitivity → Negative Reciprocity → Prosocial Behavior (effect size: −0.05; 95% CI: [−0.08, −0.03]). The confidence intervals of all the pathway do not include zero, indicating a significant effect.

**Table 5:** Mediation effects analysis of moral disengagement and negative reciprocity.

Pathway	Effect size	Boot SE	95% CI
Total mediating effect	−0.08	0.02	[−0.12, −0.05]
Moral sensitivity → moral disengagement → prosocial behavior	−0.02	0.01	[−0.05, −0.01]
Moral sensitivity → moral disengagement → negative reciprocity → prosocial behavior	−0.01	0.004	[−0.09, −0.003]
Moral sensitivity → negative reciprocity → prosocial behavior	−0.05	0.01	[−0.08, −0.03]

### Examination and analysis of integrated model M3

**Figure 1:**
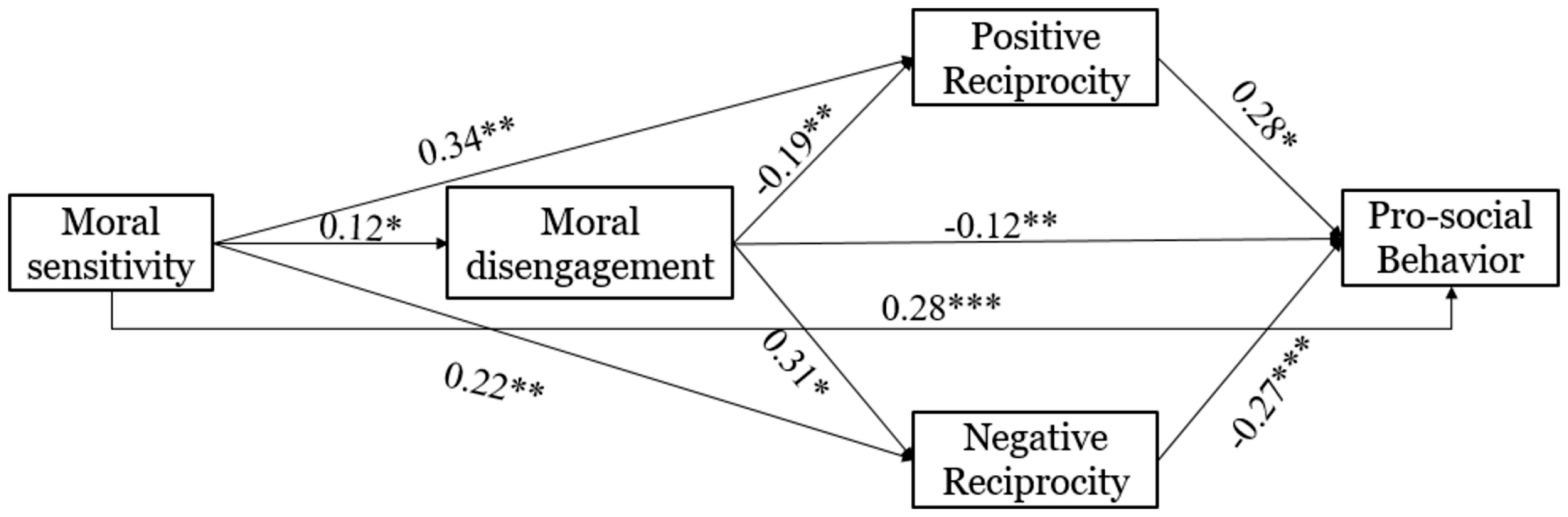
The mediating model.

To further explore the multiple mediation effects of moral disengagement and reciprocity norms in the relationship between moral sensitivity and prosocial behavior, we constructed Structural Equation Model (SEM) M3 using Amos 25.0. In this model, moral sensitivity serves as the independent variable, while moral disengagement, positive reciprocity, and negative reciprocity act as mediator variables, with prosocial behavior as the dependent variable. The model is illustrated in Figure 1. The model’s fit indices (χ^2^/df = 4.137; CFI = 0.991; RFI = 0.887; GFI = 0.997; RMSEA = 0.074) meet acceptable standards, indicating a good fit and thus validating the proposed multiple mediation model.

## Discussion

Through an investigation involving 567 college students, the study found that moral sensitivity positively predicted prosocial behavior both directly and through the mediating factor of positive reciprocity. Conversely, moral sensitivity negatively predicted prosocial behavior through moral disengagement and negative reciprocity, respectively. Additionally, significantly negative mediating effects were observed in the relationship between moral sensitivity and prosocial behavior, mediated by moral disengagement via positive or negative reciprocity.

Firstly, the study found a significant positive correlation between moral sensitivity and prosocial behavior, thereby confirming Hypothesis 1 (H1). This result is consistent with the four-component model of moral psychology and aligns with previous research (Xiang et al., 2022). Moral sensitivity, defined as a heightened awareness and interpretative tendency based on moral values (Zhao and Kushnir, 2023), leads individuals to perceive social information through a moral lens, eliciting stronger cognitive and emotional responses in moral situations (Gao and Mao, 2024). Moreover, moral sensitivity reflects an individual’s level of virtue. Those who perceive and are inspired by virtue are more likely to engage in and commit to prosocial behavior (Li and Hu, 2023).

Secondly, the study supports Hypothesis 2 (H2) by demonstrating that moral disengagement mediates the relationship between moral sensitivity and prosocial behavior. This finding aligns with moral disengagement theory, which suggests that self-regulation mechanisms can promote prosocial behavior. However, individuals with high moral sensitivity might experience impaired self-control, leading them to selectively disregard self-regulation processes. Consequently, they may avoid significant guilt or self-reproach for failing to engage in moral behavior (Gutzwiller-Helfenfinge, 2015). Zhou et al. (2024) further argue that individuals with high moral sensitivity might rationalize or deny responsibility for prosocial actions due to perceived costs, opting instead for alternative behaviors. This is consistent with previous research showing that lower levels of moral disengagement are linked to higher levels of prosocial behavior (Killer et al., 2019). Moral disengagement can be viewed as a cognitive bias that enables individuals to deny responsibility for immoral actions, thereby suppressing prosocial behavior. Lower moral disengagement facilitates self-reflection on attitudes and behaviors, encouraging prosocial actions. In contrast, higher levels of moral disengagement can obscure behavioral outcomes and lead to more immoral actions. Therefore, reducing moral disengagement is a crucial factor in fostering prosocial behavior among college students.

Additionally, the study confirms Hypothesis 3 (H3), demonstrating that moral sensitivity influences prosocial behavior through reciprocity norms. Moral sensitivity enhances individuals’ moral cognition and emotional responses, which in turn shape their reciprocity norms in social interactions. Individuals with high moral sensitivity are more perceptive of moral issues in social contexts and react accordingly (Sparks, 2015). When considering long-term exchanges and relationship balance in interpersonal interactions, people tend to reciprocate in kind, mirroring the behaviors others have shown toward them (Valle et al., 2018). The impact of reciprocity norms on prosocial behavior varies: individuals with positive reciprocity norms are more likely to engage in prosocial actions driven by feelings of gratitude, whereas those with negative reciprocity norms may focus on negative behaviors, leading to emotions such as anger that inhibit prosocial behavior. Thus, reciprocity norms are crucial in mediating the relationship between moral sensitivity and prosocial behavior.

The findings of the study also support Hypothesis 4 (H4), indicating that moral disengagement and reciprocity norms serve as chain mediators between moral sensitivity and prosocial behavior. When individuals with high moral sensitivity perceive the costs of engaging in prosocial behavior as significant, they may resort to moral disengagement to comply with reciprocity norms, which can lead to a focus on negative interpersonal behaviors. College students, who are in a crucial phase of developing stable values, may encounter social issues or exhibit immoral behaviors influenced by negative reciprocity norms (Stijnen and Dijker, 2011). In contrast, individuals with lower levels of moral disengagement are more likely to embrace positive reciprocity norms. This tendency enhances long-term social interactions, improves self-perceptions, and reduces mistaken beliefs about immoral behavior, thereby fostering prosocial actions. The emergence of prosocial behavior among college students is influenced by their moral cognition, emotions, and reciprocity tendencies in response to both cognitive and emotional factors.

The study still has limitations. First, the cross-sectional design of this study limits the ability to explore the dynamic interactions between moral sensitivity and prosocial behavior. An individual’s moral sensitivity can influence the development of prosocial behavior, which in turn can enhance moral sensitivity over time. Future research should employ longitudinal study designs to explore these dynamics in greater depth. Secondly, this study examined the impact of moral sensitivity on prosocial behavior from a single perspective. Moral sensitivity influences attentional biases, memory biases, and interpretative biases related to moral components, and may also interact with emotions such as empathy, guilt, and gratitude (Zhao and Kushnir, 2023). Future research could integrate cognitive variables (such as attention bias and memory bias) and emotional variables (such as empathy and gratitude) to further explore the mechanisms and boundary conditions of moral sensitivity’s influence on prosocial behavior, contributing to the development of interventions and educational strategies for enhancing moral sensitivity.

Despite its limitations, the study presents several advantages. Firstly, it reveals that moral sensitivity is significantly and positively correlated with moral disengagement, positive reciprocity, negative reciprocity, and prosocial behavior. Secondly, the study demonstrated that moral sensitivity influences prosocial behavior through the mediating roles of moral disengagement and reciprocity norms, as well as the chain-mediating role of both moral disengagement and reciprocity norms. This research underscores the intricate roles of moral disengagement and reciprocity norms in amplifying prosocial behavior through moral sensitivity. These insights are crucial for developing and promoting prosocial behavior norms among college students.

## Conclusion

Moral sensitivity is significantly positively correlated with moral disengagement, positive reciprocity, negative reciprocity, and prosocial behavior. Moral disengagement is significantly negatively correlated with positive reciprocity and prosocial behavior, and significantly positively correlated with negative reciprocity; Prosocial behavior is significantly positively correlated with positive reciprocity and negatively correlated with negative reciprocity.

Moral disengagement and reciprocity norms played multiple mediating roles between moral sensitivity and prosocial behavior. Specifically, moral sensitivity affected prosocial behavior through the independent mediating effect of moral disengagement, reciprocity norms, as well as the chain mediating effect of moral disengagement and reciprocity norms.

## Data Availability

The raw data supporting the conclusions of this article will be made available by the authors, without undue reservation.
